# Activation of apoptosis in hepatocellular carcinoma by the Chinese traditional medicine Hu Qisan

**DOI:** 10.3892/etm.2012.862

**Published:** 2012-12-18

**Authors:** XIANGJUN ZENG, XIANGLI LI, XIAOWEI XUE, ZHENG-MING SHI, PING FENG, PENGYAN WANG, XUE-JIANG WANG

**Affiliations:** 1 Pathophysiological Department, Capital Medical University, Beijing 100069;; 2 Department of General Surgery, Beijing Jishuitan Hospital, Beijing 100035, P.R. China

**Keywords:** Hu Qisan, hepatocellular carcinoma, HtrA serine peptidase 2, apoptosis

## Abstract

To investigate the effects of Hu Qisan (HQS) on apoptosis in diethylnitrosamine (DEN)-induced hepatocellular carcinoma (HCC), a Solt-Farber two-step test model of precancerous liver lesions was established in rats using a previously described method. HQS (4 and 8 g/kg body weight/day) was administered for 4 weeks, after the majority of the liver was removed. HepG2 cells were used to detect the HtrA serine peptidase 2 (HtrA2/Omi) release from mitochondria and caspase-3 activation promoted by HQS. Exposure of the rats to DEN for 6 weeks induced hepatic carcinogenesis. HQS (4 and 8 g/kg body weight/day) markedly induced cell apoptosis. The protective effects against hepatic carcinogenesis were mediated by multiple mechanisms, including the reduction of DEN-induced γ-GT-positive cell proliferation, mitochondrial morphological changes, HtrA2/Omi release from mitochondria and the activation of caspase-3. In conclusion, HQS is a potential anti-carcinogenic agent that may induce apoptosis by reducing the inhibitory effects of X-linked inhibitor of apoptosis protein (XIAP) on caspase-3. Thus, HQS should be further explored as a potentially promising new therapeutic agent against human hepatic cancer.

## Introduction

A large number of factors, receptors and downstream elements in cell signaling cascades regulate the proliferation and apoptosis of hepatic carcinoma. Dysregulation of the balance between these processes represents a pro-tumorigenic principle in human hepatic carcinogenesis, where there is usually activation of proliferative signals and an inhibition of death processes, leading to the survival and subsequent proliferation of carcinoma cells. Apoptosis represents a physiological method for eliminating excess cells during liver development and regeneration (1). Insufficient apoptosis has been associated with the development and progression of tumors in the liver and biliary tree (1). Hepatocellular carcinoma (HCC) is the fifth most frequent neoplasm worldwide and the third leading cause of cancer-related mortality. To date, systemic chemotherapeutic treatment is ineffective against HCC (2), in part due to the resistance to apoptosis that is observed in HCC cells.

Apoptotic signaling within the cell is mainly transduced via two molecular pathways: the death receptor pathway (also called the extrinsic pathway) and the mitochondrial pathway (also called the intrinsic pathway) (1). However, molecular alterations have been reported for HCC that alter its apoptotic response, including the p53 and transforming growth factor (TGF)-β pathways. Therefore, researchers have been committed to finding ways to promote apoptosis, independent of the altered molecules in HCC. The intrinsic pathway is triggered by various extra or intracellular signals that induce mitochondrial dysfunction, resulting in altered membrane permeability and the release of mitochondrial proteins into the cytosol, including the pro-apoptogenic factors cytochrome *c* and HtrA serine peptidase 2 (HtrA2/Omi) (3). Once this intrinsic pathway is activated, hepatic carcinogenesis may be restricted; therefore, these proteins are also targets in cancer research. Hu Qisan (HQS), a Chinese traditional medicine, has notable therapeutic effectiveness against hepatocirrhosis as well as the ability to block and reverse hepatocarcinogenesis. Our previous study confirmed that HQS has a clear effect on hepatocarcinogenesis (4). In the present study, we used HQS to activate mitochondrial-controlled apoptosis in HCC. In addition, the components of HQS that contribute to the proapoptotic process are discussed.

## Materials and methods

### 

#### Preparation of Hu Qisan

HQS was created from 8 medicinal herbs containing glycoprival granules. The herbal drugs, including *Ramulus Visci*, *Radix Astragali seu Hedysari*, *Radix Curcumae* and *Radix Salviae Miltiorrhizae*, were gently boiled in distilled water for 60 min to reduce the volume. The herbal mixture was soaked for 1 h before boiling. The decoction was filtered through delipidated gauze and then concentrated to an ointment through decompression treatment. The ointment was dried in a vacuum to form extractum, which was ground. The powdered preparation was stored in a refrigerator until subsequent use.

### Preparation of components of Hu Qisan

#### Mistletoe alkali

Mistletoe was crushed to small particles, 1 kg of which was immersed into various acidic aqueous solutions (1.5, 1.0 and 0.5% HCl) for 48 h. The ratio of particles to HCl was 20:1. Then, the solutions were distilled 3 times for 2 h at 50°C. The solutions were filtered and centrifuged for extraction of the supernatant. The concentrated supernatants (extractum) were dissolved in 2% HCl, which was centrifuged twice to extract the supernatant. Chloroform was used to remove the lipid from the supernatant solution and obtain the total alkali A. Normal butyl alcohol was used to extract the total alkali B from the supernatant solution. Total alkali A was mixed with total alkali B to produce the total alkali used in the following experiments.

#### Mistletoe polysaccharide

Powder crushed from dried mistletoe (1 kg) was dissolved in water at a ratio of 1:28, powder to water (weight/v). After 3 h in a 95°C water bath, the polysaccharide was dissolved in water. Then, the solvent was filtered and centrifuged at 5,867 × g to remove the dregs. In order to condense the polysaccharide solvent, 95% ethanol was added to the solvent to yield 80% ethanol dissolved in polysaccharide solvent. The solvent was incubated at 4°C overnight and the precipitate at the bottom was dissolved in water. In order to remove the protein from the solvent, 50% trichloroacetic acid was added to yield a final concentration of 10% trichloroacetic acid and was centrifuged at 1,467 × g to remove the precipitate. The pH was adjusted to 7.0. To obtain the polysaccharide powder, 95% ethanol was then added, followed by centrifugation at 1,467 × g. The precipitate was recovered and acetone was added, followed by evaporation to remove the water and ethanol. We used ion-exchange chromatography to purify the polysaccharide at a neutral location. After qualitative and quantitative analysis, the purified polysaccharide included 12% neutral arabinogalactan and a small amount of xylose glycan. The extraction efficiency was 3.98%. The polysaccharide powder was dissolved in saline.

#### Animals and treatment

Male Wistar rats (6 weeks old) weighing 135–149 g were purchased from the Animal Department of Capital Medical University. The experiment was performed as previously described (5), using the Solt-Farber two-step test model of precancerous liver lesions. Diethylnitrosamine (DEN; 200 mg/kg) was injected into the abdominal cavity of experimental rats as an initiating agent. After 2 weeks, we performed a selective promoting procedure [feeding with a diet containing 0.015% 2-acetyl aminofluorene (2-AAF)], which lasted for 6 weeks. The majority of the liver was removed from the rats at the end of the third week. Sixty rats were allowed free access to a pellet diet and water and were divided into four groups: model group, high-dose therapeutic group (8 g/kg/day HQS), low-dose therapeutic group (4 g/kg/day HQS) and a 5-fluorouracil (5-FU) group (250 mg/day on days 1–5 of each week for 4 weeks). Rats in the therapeutic groups began the treatment with HQS 1 week after the majority of the liver was removed, for 4 weeks. Rats in the normal group were fed normally. After 8 weeks, all rats were sacrificed under anesthesia with pentobarbital after 24 h fasting. Hepatic tissue was taken from the right anterior, right back and caudal lobe.

#### Histochemical staining for γ- GTase-positive foci

γ-Glutamyl-transpeptidase isoenzyme (γ-GTase)-positive foci were determined as previously described (6). Fresh liver tissue was cut into 8-*μ*m-thick sections, which were mounted on slices and air-dried. Fresh solution containing γ-glutamyl-4-methoxy-2-naphthylamide (GMNA), γ-GTase, azo-coupling dye, Fast blue BBN (diazotized 4′-amino-2′,5′-diethoxybenzanilide), 0.1 mol/l Tris buffer (pH 7.4) and saline was prepared and pipetted onto the sections which were then incubated in the dark for 30–45 min. Following incubation, the slices were rinsed in saline for 2 min and transferred to 0.1 mol/l cupric sulphate solution for 2 min. The slices were rinsed in saline followed by distilled water. The sections were air-dried, placed into 10% glycerol and observed under a light microscope. Four sections from each liver were examined under 5 fields. The γ-GTase-positive foci counted were divided according to various size ranges and the results were expressed as the percentage of foci area in sections. Sections of kidney tissue served as positive controls for γ-GTase staining.

#### Isolation of mitochondrial and cytosolic fractions from HepG2 cells

HepG2 cells were cultured with 10% fetal bovine serum (FBS) in Dulbecco’s modified Eagle’s medium (DMEM). HQS at a high concentration (3 mg/ml) and low concentration (1.5 mg/ml), mistletoe polysaccharide (25 mg/ml), mistletoe alkali (20 mg/ml) and 5-FU (1 *μ*g/ml) were administered to promote apoptosis of the cells for 12 h to detect HtrA2/Omi release from the mitochondria and for 24 h to detect caspase-3 activation.

Mitochondrial and cytosolic fractions were isolated to detect HtrA2/Omi distribution using the mitochondria isolation kit for cultured cells (89874; Pierce, Rockford, IL, USA). Briefly, reagents were added in order and centrifuged, the cytosolic fraction was removed and the pellet containing the isolated mitochondria was washed and centrifuged again. Lysis buffer was added and centrifuged to obtain the mitochondrial protein.

#### Western blotting of protein expression

Following treatment, 40 *μ*g cell lysate was subjected to sodium dodecyl sulphate-polyacrylamide gel electrophoresis (SDS-PAGE). The gel was transferred to a polyvinylidene fluoride (PVDF) membrane (Millipore, Bedford, MA, USA), after which the membrane was blocked with 5% BSA/0.05% Tween-20/phosphate-buffered saline (PBS), followed by overnight incubation at 4°C with primary antibody diluted in 5% BSA/0.05% Tween-20/PBS. The primary antibodies used were as follows: rabbit anti-HtrA2/Omi, goat anti-cleaved caspase-3 (1:750 dilution; Cell Signaling Technology, Danvers, MA, USA), mouse anti-voltage dependent anion channel (VDAC; Cell Signaling Technology) and mouse anti-β-actin (Sigma, Munich, Germany). The membrane was washed 3 times with 0.05% Tween-20/PBS and incubated with alkaline phosphataseconjugated secondary antibody (Cell Signaling Technology) at a 1:2,000 dilution (5% BSA/0.05% Tween-20/PBS; 1 h at room temperature). The membrane was then rewashed 3 times and developed by the addition of SuperSignal West Pico chemiluminescent substrate (Cat: 34077; Thermo Scientific, Rockford, IL, USA). The membrane was scanned and analyzed by densitometry using Adobe Photoshop (Adobe Systems Inc., San Jose, CA, USA) and Image J (NIH, Bethesda, MD, USA). Signal intensities of the activated proteins were normalized to β-actin or VDAC.

#### Reverse transcription-polymerase chain reaction (RT-PCR) protocol

The reverse transcription system and PCR Master mix were purchased from Promega (Madison, WI, USA). The primers for HtrA2/Omi (F, 5′-TGTGTTCTTCAGAGCCCAG GACTGC-3′; R, 5′-CTACAGCT CCGAGAGCCAAGTTTCC-3′) and β-actin (F, 5′-TGGAATCCTGTGGCATCCATGAAAC-3′; R, 5′-TAAAACGCAGCTCAGTAACAGTCCG-3′) were synthesized by Genecore (Shanghai, China). RNA was extracted from the tissues using TRIzol reagent (Invitrogen, Carlsbad, CA, USA). After denaturing at 94°C for 1 min, the PCR reaction consisted of 30 cycles (HtrA2/Omi and β-actin) of denaturation at 94°C for 30 sec, annealing at 60°C for 40 sec and elongation at 72°C for 45 sec, followed by one cycle of final extension at 72°C for 5 min. A negative control without template cDNA was always included. The expected sizes of the PCR products were 402 bp for HtrA2/Omi and 320 bp for β-actin and all PCR products were analyzed by 2% agarose gel electrophoresis with ethidium bromide staining. The optical density (OD) of the expected bands was measured and the OD ratio of HtrA2/Omi mRNA to β-actin mRNA was used to determine the relative amount of HtrA2/Omi/β-actin.

#### Statistical analyses

Comparisons were made using one-way analysis of variance (ANOVA). All experiments were repeated at least 3 times. P<0.05 was considered to indicate a statistically significant difference. Data are presented as the means ± standard error of the mean.

## Results

### 

#### DEN and liver resection promoted carcinogenesis in vivo

**Figure 1.**
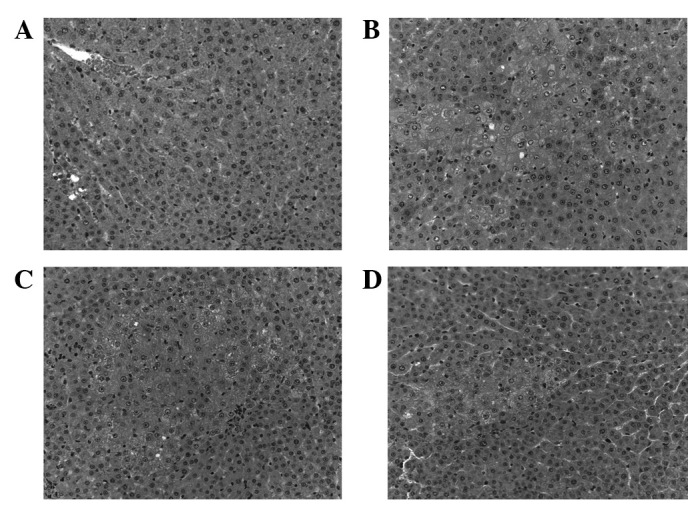
Representative sections of hepatocellular carcinoma (HCC) stained by hematoxylin and eosin (H&E). (A) Section from the liver of a Wistar rat. (B) Section from the liver of a diethylnitrosamine (DEN) and 0.015% 2-acetyl aminofluorene (2-AAF) treated rat (HCC model). (C) Section from the liver of a HCC rat treated with Hu Qisan (HQS; 8 g/kg body weight/day). (D) Section from the liver of a HCC rat treated with HQS (4 g/kg body weight/day).

**Figure 2.**
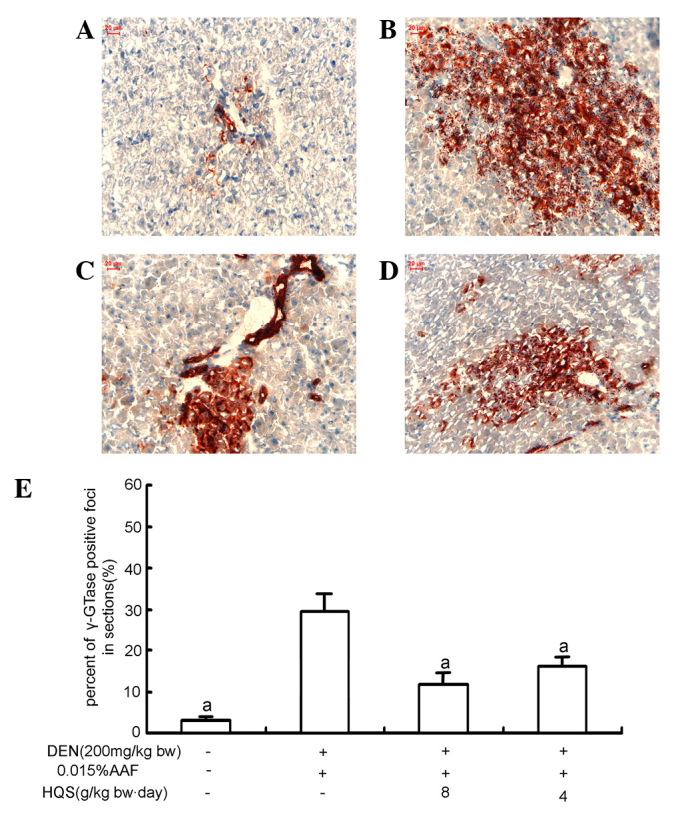
Representative γ-GTase staining in liver sections from four groups. (A) Normal liver sections from a Wistar rat with γ-GTase expression around the portal areas. (B) Liver section from a carcinogenic model rat with γ-GTase expression scattered in the hepatic tissue. (C) HQS (8 g/kg body weight/day) treated rat with γ-GTase expression reduced to the portal areas. (D) HQS (4 g/kg body weight/day) treated rat with less γ-GTase expression than the model group. (E) Percentage of area occupied by foci in the sections A–D. n=6; ^*^P<0.05 vs. sections from the carcinogenic model group. γ-GTase, γ-glutamyl transpeptidase; DEN, diethylnitrosamine; 2-AAF, 2-acetyl aminofluorene; HQS, Hu Qisan.

H&E and γ-GTase staining were used to demonstrate that DEN and liver resection promotes carcinogenesis in rats. In the control group, the hepatic lobules were clear and intact; and the liver cells were polygonal and radially arranged around the central vein (Fig. 1A). γ-GTase staining demonstrated little γ-GTase expression in the portal areas of normal rat liver (Fig. 2A). In the DEN and liver resection group, hepatic cells demonstrated early HCC transformation. The normal hepatic cord structure and sinusoids were damaged. Cells proliferated to form foci containing basophilic or acidophilic cells. A number of these cells invaded surrounding tissues and some cloudy cells were slightly larger with prominent nucleoli. γ-GTase staining showed that γ-GTase-positive foci were brown/red and scattered in the hepatic tissue. At the edge of γ-GTase-positive foci, hepatic cells were suppressed by proliferating cells (Fig. 2B).

#### HQS treatment attenuated precancerous lesions in rat liver

Following treatment with HQS, carcinogenesis in hepatic tissues triggered by DEN and liver resection was partly reversed or blocked. H&E staining revealed that the number of proliferating foci significantly decreased and the number of enlarged nuclei with prominent nucleoli also decreased (Fig. 1C and D). γ-GTase staining showed that the size of γ-GTase positive foci was smaller and the number of suppressed hepatic cells was attenuated (Fig. 2C–E).

#### HQS upregulated HtrA2/Omi expression in precancerous lesions in rat liver

**Figure 3.**
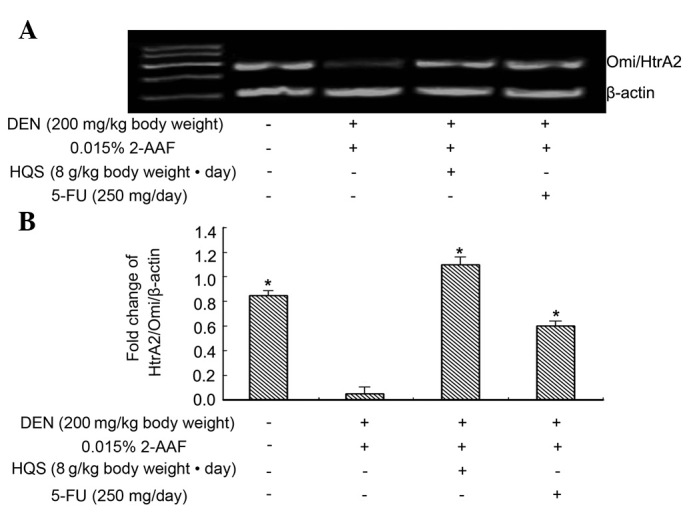
Reverse transcription-polymerase chain reaction analysis of HtrA2/Omi mRNA and quantification of HtrA2/Omi mRNA. (A) HtrA2/Omi mRNA and (B) HtrA2/Omi normalized against β-actin. Data are the mean ± standard error of the mean (SEM; n=4/group). ^*^P<0.05, compared with the group treated with DEN and 2-AAF. DEN, diethylnitrosa-mine; 2-AAF, 2-acetyl aminofluorene; HQS, Hu Qisan; 5-FU, 5-fluorouracil.

RT-PCR results revealed that HtrA2/Omi is expressed in normal and carcinogen-treated hepatic tissues and its expression in carcinogen-treated hepatic tissues is much lower than in normal tissues. Used as a control to prevent carcinogenesis, 5-FU did not change the expression of HtrA2/Omi in the tissues exposed to carcinogen, while HQS significantly increased its expression (Fig. 3).

**Figure 4.**
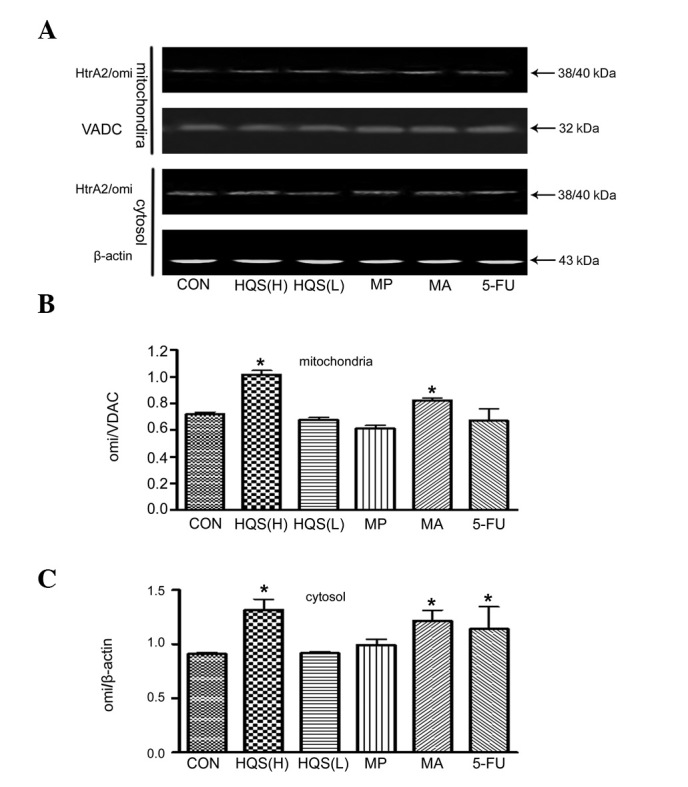
Effect of HQS on HtrA2/Omi release in HepG2 cells. HtrA2/Omi content in mitochondrial and cytoplasmic compartments was evaluated using western blotting. Protein expression was corrected according to the loading control of VDAC and β-actin in mitochondria and cytosolic fractions, respectively. (A) Western blotting gel shows the HtrA2/Omi contents of mitochondrial and cytoplasmic compartments with different treatments, as indicated. (B) Summarized data from experiments in (A). High-dose HQS significantly increased the HtrA2/Omi content of the mitochondria, as did its component mistletoe alkali. 5-FU, as a control to prevent carcinogenesis, diminished the HtrA2/Omi content in the mitochondria. (C) Summarized data from experiments in (A). High-dose HQS significantly increased HtrA2/Omi release from the mitochondria and its component mistletoe alkali increased its release to the cytoplasm effectively. As a control to prevent carcinogenesis, 5-FU promoted HtrA2/Omi release into the cytoplasm. n=5; ^*^P<0.05 compared with untreated HepG2 cells. HQS, Hu Qisan; HQS(H), HQS at 8 g/kg body weight/day; HQS(L), HQS at 4 g/kg body weight/day; MP, mistletoe polysaccharide; MA, mistletoe alkali; VADC, voltage-dependent anion channel; 5-FU, 5-fluorouracil.

**Figure 5.**
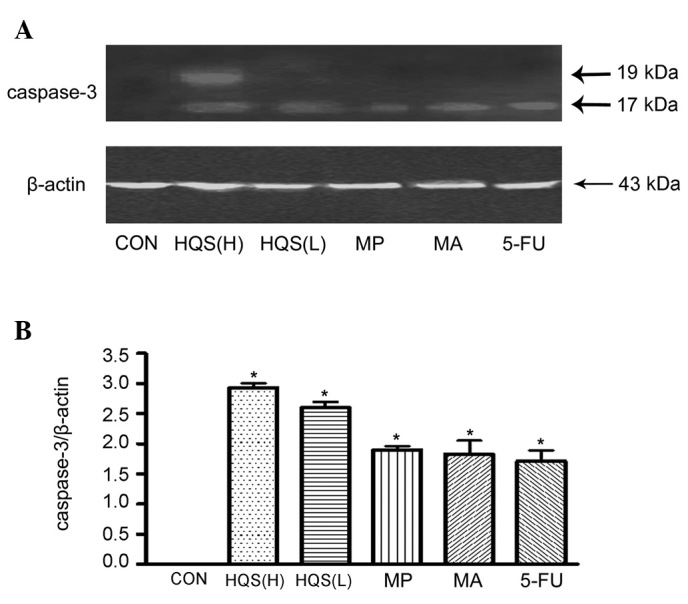
Effects of HQS on caspase-3 activity in HepG2 cells. HepG2 cells were treated with DEN for 24 h. Caspase-3 activity was determined using an antibody against cleaved caspase-3. (A) Western blotting shows caspase-3 contents in the cytoplasm with different treatments, as indicated. (B) Summarized data from experiment (A). n=3; ^*^P<0.05 compared with untreated HepG2 cells. HQS, Hu Qisan; HQS(H), HQS at 8 g/kg body weight/day; HQS(L), HQS at 4 g/kg/day; MP, mistletoe polysaccharide; MA, mistletoe alkali; DEN, diethylnitrosamine; 5-FU, 5-fluorouracil.

HQS stimulated HtrA2/Omi release from mitochondria and caspase-3 activation. Consistent with the apoptotic nature of DEN-induced neuronal carcinogenesis, there was little HtrA2/Omi release from the mitochondria into the cytosol in the control HepG2 cells. HQS in high doses promoted HtrA2/Omi release from the mitochondria to the cytosol, as measured by western blot analysis (Fig. 4A and C). The HtrA2/Omi levels in the mitochondria were higher than in the control HepG2 cells (Fig. 4A and B) following HQS treatment, which confirms the HtrA2/Omi mRNA upregulation by HQS in the tissues exposed to carcinogen. HtrA2/Omi also substantially promoted caspase-3 activation following the onset of HQS treatment (Fig. 5).

**Figure 6.**
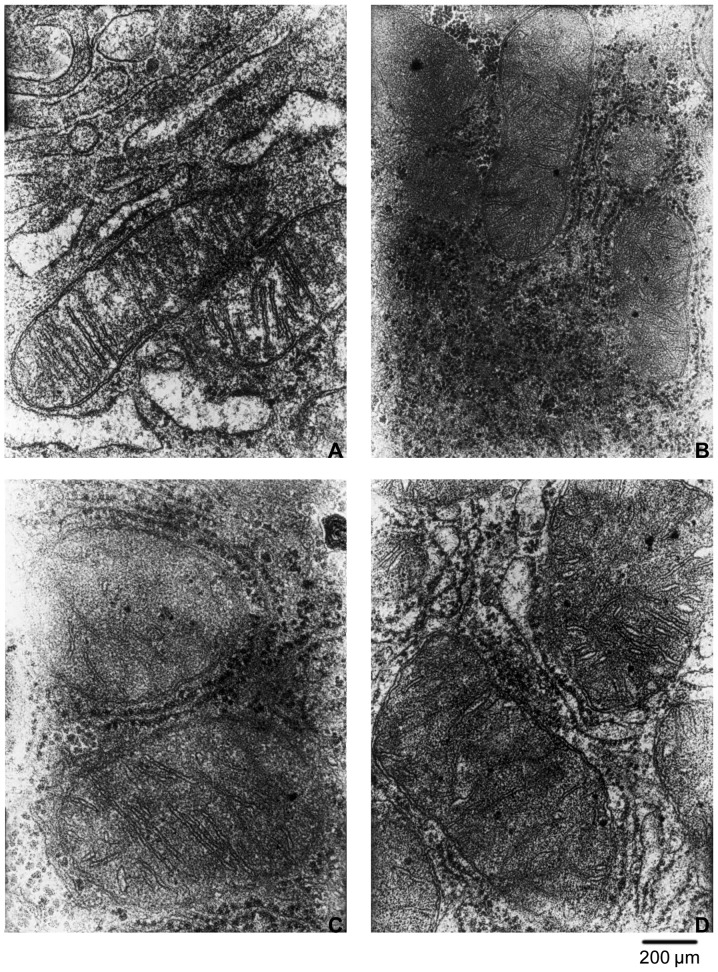
Electronic microscopic analysis of mitochondrial changes in liver tissues. (A) Mitochondria from the liver tissue of a Wistar rat. (B) Mitochondria from hepatocellular carcinoma (HCC). (C) Mitochondria from HCC treated with Hu Qisan (HQS) at 8 g/kg body weight/day. (D) Mitochondria from HCC treated with HQS at 4 g/kg body weight/day.

Consistent with the above observations, HQS induced a change in the number and morphology of mitochondria 4 weeks after treatment. At 8 g/kg body weight, HQS treatment increased the number and size of mitochondria in HCC, decreased the number of crista in mitochondria and the mitochondrial membrane was not intact. At 4 g/kg body weight, HQS showed no significant change in the number and morphology of mitochondria (Fig. 6).

## Discussion

Cancer occurs or progresses because malignant cells fail to undergo apoptosis, either spontaneously or in response to chemotherapy. Failure to activate caspases may account for this resistance to apoptosis. Many strategies for restoring apoptosis sensitivity in refractory cancers have been tested (7–9) and some are undergoing clinical testing in humans. A potential advantage of inhibitors of apoptosis proteins (IAPs) as drug targets is that they operate at distal points in apoptotic pathways, potentially bypassing many upstream defects in apoptosis-regulatory mechanisms in tumors (10). However, this strategy is only valid if tumor and normal cells demonstrate differential sensitivities to IAP suppression.

In this study, we describe HQS, a traditional Chinese medicine and inhibitor of pre-hepatocarcinoma, as was determined from studies using the classical preneoplastic marker enzyme for hepatic chemical carcinogenesis, γ-GTase. γ-GTase is a cell surface enzyme that initiates the cleavage of extracellular glutathione and glutathione conjugates. Hydrolysis of glutathione in glomerular filtrate and fluids in other ducts and glands throughout the body provides a mechanism for the body to retain amino acids containing glutathione. In the absence of γ-GTase-initiated cleavage, glutathione is excreted from the body, resulting in fatal cysteine deficiency. In rodents, γ-GTase is a single copy gene whose expression is regulated in a developmental and tissue specific manner. Several γ-GTase genes or pseudogenes are present in humans. γ-GTase activity is elevated in carcinogen-induced tumors of animals. Common human epithelial tumors, including breast, ovarian and prostate tumors, are γ-GTase-positive. Synthesis of γ-glutamyl pro-drugs is used as a new approach in chemotherapeutic drugs to treat γ-GTase-positive tumors. The placenta form of γ-GTase is hardly detectable in normal rat liver and is markedly induced in liver-bearing foci and nodules (11). Our histochemical data demonstrated that DEN exposure formed γ-GTase foci which are correlated with the development of HCC. However, HQS at different doses prevented the formation of γ-GTase foci (Fig. 1). Since the synthesis of γ-glutamyl prodrugs is used as a new approach for targeting γ-GTase-positive tumors, the effect of HQS on preventing the formation of γ-GTase foci in liver indicates that it is capable of protecting against DEN-induced hepatocarcinogenesis.

X-linked inhibitor of apoptosis protein (XIAP) is the best studied protein in the human IAP family of proteins from the standpoint of biochemical mechanism (12) and its overexpression in several types of human cancers has been documented (13–16). XIAP suppresses the downstream proteases, including caspase-3 and -7, via its BIR2 region (17–19). The objective of this study was to demonstrate that HQS overcomes the inhibitory effects of XIAP on proteases in hepatocarcinoma cells and tissues. Our study indicates that HQS inhibits XIAP by promoting HtrA2/Omi expression and release from mitochondria in hepatocarcinoma. Two HQS components were responsible for inducing HtrA2/Omi release from the mitochondria. Moreover, a perfect correlation was observed between HtrA2/Omi release and induction of caspase-3 activation in HepG2 cells. Cancer cells have an intrinsic drive to activate caspases while upregulated IAP expression counteracts it (20). Therefore, functional removal of IAPs permits apoptosis to occur in tumor cells but not in normal cells (20). This is consistent with evidence of processed caspase-3 in tumor cell lines and tumor tissues being offset by the overexpression of XIAP or other IAP family members (20). In this regard, many causes of caspase activation in tumor cells may be envisioned, including protooncogene activation, disobeyance of cell cycle checkpoints associated with defective DNA replication and chromosome segregation and loss of cell attachment (anchorage-independent growth), all of which are known to drive apoptosis unless countered by anti-apoptotic proteins (21). By contrast, normal cells are expected to have less drive to caspase activation, thus rendering them less dependent on IAPs; a theory supported by gene ablation studies in mice, which have revealed little or no adverse phenotypes in animals lacking XIAP, cIAP1, cIAP2, or neuronal apoptosis inhibitory protein (NAIP) (22,23)

HtrA2/Omi has a dual role as a caspase activator via inhibition of IAP proteins and as an effector of necrosis-like programmed cell death through its serine protease activity (24,25). In our study, cytosolic release of HtrA2/Omi was enhanced by HQS (Fig. 4C) in HepG2 cells. Moreover, levels of HtrA2/Omi mRNA and HtrA2/Omi in the mitochondria were elevated by HQS (Figs. 3 and 4B) in HepG2 cells. These results suggest that HQS targets XIAP to activate caspases, which then induce apoptosis in hepatic cancer cells.

XIAP is the most studied of the human IAP family members from the standpoint of biochemical mechanisms and its overexpression in several types of human cancers has been documented. XIAP suppresses the downstream effector protease, caspase-3. Our results demonstrate the inhibitory effect of HQS on XIAP and its downstream proteins (Fig. 5). In addition, HQS is able to suppress the proliferation of γ-GTase-positive (Fig. 2) cells in hepatic tissues. Therefore, HQS may be a potential anti-carcinogenic agent through its inhibitory action on the XIAP pathway.

In conclusion, results from this study clearly revealed that HQS at high and low doses is able to promote HtrA2/Omi expression and release from mitochondria in HCC. The components responsible for this effect are identified as mistletoe alkali and mistletoe polysaccharide. Our findings support the use of HQS as antitumor medicine to promote apoptosis, which was inhibited by XIAP in cancer cells.
